# Potential Involvement of Type I Interferon Signaling in Immunotherapy in Seasonal Allergic Rhinitis

**DOI:** 10.1155/2016/5153184

**Published:** 2016-12-19

**Authors:** Lina Mattson, Antonio Lentini, Danuta R. Gawel, Tejaswi V. S. Badam, Mikael Benson, Torbjorn Ledin, Colm E. Nestor, Mika Gustafsson, Jordi Serra-Musach, Janne Bjorkander, Zou Xiang, Huan Zhang

**Affiliations:** ^1^The Centre for Personalized Medicine, Department of Clinical and Experimental Medicine, Division of Pediatrics, Linkoping University, Linkoping, Sweden; ^2^Bioinformatics, Department of Physics, Chemistry and Biology, Linkoping University, SE-581 83 Linkoping, Sweden; ^3^Division of Neuro and Inflammation Science, Department of Clinical and Experimental Medicine, Linkoping University, Linkoping, Sweden; ^4^Department of Otorhinolaryngology in Linkoping, Anaesthetics, Operations and Specialty Surgery Center, Region Ostergotland, Sweden; ^5^Futurum-Academy for Health and Care, County Council of Jonkoping, Jonkoping, Sweden; ^6^Department of Health Technology and Informatics, Faculty of Health and Social Sciences, The Hong Kong Polytechnic University, Hung Hom, Kowloon, Hong Kong

## Abstract

Specific immunotherapy (SIT) reverses the symptoms of seasonal allergic rhinitis (SAR) in most patients. Recent studies report type I interferons shifting the balance between type I T helper cell (Th1) and type II T helper cells (Th2) towards Th2 dominance by inhibiting the differentiation of naive T cells into Th1 cells. As SIT is thought to cause a shift towards Th1 dominance, we hypothesized that SIT would alter interferon type I signaling. To test this, allergen and diluent challenged CD4^+^ T cells from healthy controls and patients from different time points were analyzed. The initial experiments focused on signature genes of the pathway and found complex changes following immunotherapy, which were consistent with our hypothesis. As interferon signaling involves multiple genes, expression profiling studies were performed, showing altered expression of the pathway. These findings require validation in a larger group of patients in further studies.

## 1. Introduction

Specific immunotherapy (SIT) is a clinically safe method to achieve tolerance in patients with allergies by repeatedly exposing them to increasing doses of a specific allergen administered either sublingually, subcutaneously [1], or intralymphatically [2]. It is thought to shift the immune response from an excessive Th2 response to a Th1 response and induce T regular cells (Tregs), which is supported by the findings of decreasing IL-4, IL-5, and IL-13, as well as increasing IL-10 [1]. Increasing levels of IFN*γ* and decreasing levels of IL4R in response to SIT [3] provide additional support to the theory. The mechanism of tolerance is, however, not fully understood.

Type II interferons, specifically interferon gamma, are typically associated with a Th1 response. Type I interferons, however, are classically known to suppress allergen- and microbial-specific Th2 responses [4] and to be required for the induction of the production of IFN*γ* during viral infections [5], but recent findings show that chronic interferon type I signaling suppresses the de novo formation of Th1 cells [6].

We sought to understand whether aberrant interferon type I signaling indirectly plays a part in the disproportionate Th2 response by suppressing the de novo formation of Th1 cells in patients with seasonal allergic rhinitis, and if so, is this rectified by sublingual immunotherapy (SLIT)?

For this purpose, we analyzed paired samples from 4 patients with SAR before and after one year of SLIT, as well as from 4 of healthy controls whom were collected simultaneously with the patient samples before treatment. The results showed that, although levels differed greatly among patients before treatment and healthy controls, after one year of SLIT, the gene expression of the patients resembled those of the controls (Principal Component Analysis, PCA). A novel strategy was developed to analyze the expression of the whole pathway as opposed to just a handful of genes, and type I interferon pathway was highlighted. The new strategy was supported by Ingenuity Pathways Analysis (IPA) of the paired patients after two years of SLIT, which indicated a SLIT-induced change of the activity of the type I interferon pathway. Because of the importance of elucidating immunotherapy-induced mechanisms of tolerance in allergic inflammation further studies in larger materials are warranted to examine the role of the type I interferon pathway.

## 2. Materials and Methods

### 2.1. Ethics Statement

A written, informed consent was obtained from all patients and healthy controls and the study was approved by the ethics committee of Linkoping University.

### 2.2. Subjects

Four patients with SAR, who were allergic to birch pollen, and four healthy controls were included. The median age of patients was 48.75 ± 6.13 and all were women, while the median age of healthy controls was 34.0 ± 1.0 and 2 were women. All patients had a positive history for birch pollen-induced SAR for at least two years. In addition, sensitivity to birch pollen was also confirmed with skin prick test with extracts from birch and 2 other pollens, 3 animals, 2 mites and 2 moulds (ALK Abelló, Hørsholm, Denmark) and by an ImmunoCap Rapid Test (Phadia, Thermo Fisher Scientific, Uppsala, Sweden), which tests for birch, grass, and house dust mite sensitivity in all subjects. All healthy controls were negative for all tested allergen sources including birch pollen and house dust mite. All patients were clinically evaluated by the same physician (JB) before, after one year, and after two years of SLIT treatment. This included contacts during or close to the pollen seasons. All patients responded favorably to treatment.

### 2.3. Allergen Challenge Assay

Peripheral blood mononuclear cells (PBMCs) obtained from 4 patients and 4 controls were challenged with diluent (D; PBS) or allergen extracts (A) from birch pollen (ALK Abelló, 100 *μ*g/mL) at a density of 10^6^ cells/mL for 7 days in RPMI 1640 supplemented with 2 mM L-glutamine (PAA Laboratories, Linz, Austria), 5% human AB serum (Lonza, Switzerland), 5 *μ*M *β*-mercaptoethanol (Sigma-Aldrich, St. Louis, Missouri, USA), and 50 *μ*g/mL gentamicin (Sigma- Aldrich, St. Louis, Missouri, USA) [7].

### 2.4. Flow Cytometry

PBMCs were collected after 7 days with or without allergen challenge (in vitro) from patients before and after SLIT as well as in healthy controls. Cell sorting was performed on a FACS Aria flow cytometer (BD Biosciences, San Diego, CA, USA) and the data was analyzed by FlowJo 7.6 (Tree Star, Inc., San Carlos, CA). Human IgG (Sigma-Aldrich, St Louis, MO, USA) at a final concentration of 200 *μ*g/mL was used to block cells prior to staining. Mouse anti-human CD4-FITC and all matched isotype controls were purchased from BD Pharmingen (San Diego, CA, USA). Mouse antihuman CD3-Pacific Blue™ and all matched isotype controls were purchased from Biolegend (San Diego, CA, USA).

### 2.5. RNA Preparation and cDNA Synthesis

The typical purity of sorted CD4^+^ T cells was >98%. Total RNA was extracted using a miRNeasy Minikit (QIAGEN, Valencia, CA, USA) according to the manufacturer's instructions. The quantity of RNA was measured with a NanoDrop ND-1000 UV Spectrophotometer (NanoDrop Technologies, Wilmington, DE, USA). cDNA synthesis was performed with a High-Capacity cDNA Reverse Transcription Kit (Allied Business Intelligence, Inc., New York, USA).

### 2.6. Gene Expression Microarray Analysis

CD4^+^ T cells were isolated using flow cytometry and the quantity and quality of RNA was examined as described before. Gene expression microarrays (Illumina, San Diego, CA, USA) were performed as previously described [8, 9].

### 2.7. Statistics

A ranked list of genes was prepared for the gene expression microarray (GEM) data by fitting the data to a linear model to compare each of the different time points. This was performed in the statistical programming language R using LIMMA package [10]. Principal component analysis (PCA) was used to obtain an overview of gene expression changes before SLIT, after one year of SLIT and controls, based on the results of the LIMMA analyses. A fold change ± 1.5 and *p* < 0.05 (Two-tailed* T*-test) were used to identify differentially expressed genes, as previously described [11]. Pathway analysis was performed using AMIGO. A *p* value <0.05 was considered significant. The list of genes of all time points of patients and controls (as described before) with their respective log fold changes and *p* values obtained was uploaded into Ingenuity Pathways Analysis (IPA) application software. Then the analysis for the upstream regulators of the significant differentially expressed genes was performed [12].

## 3. Results and Discussion

**Figure 1:**
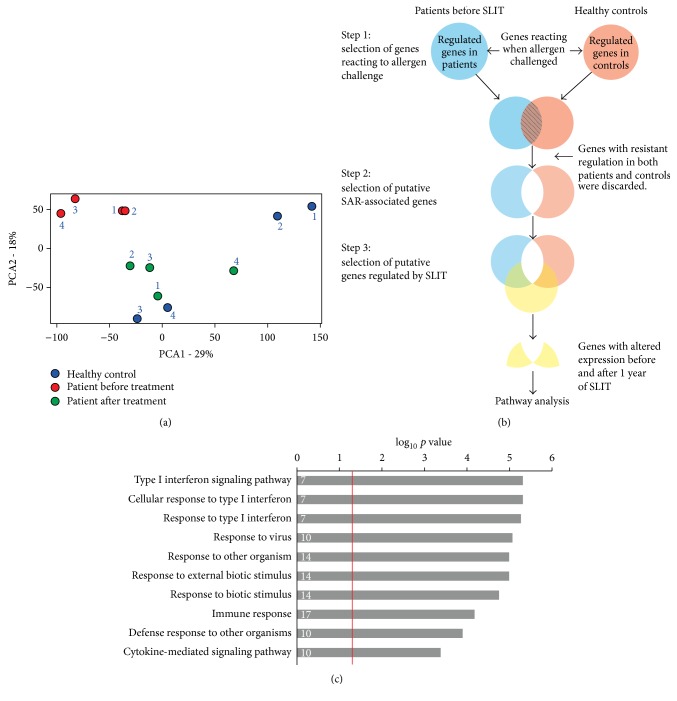
The sublingual immunotherapy alters the signaling of type I interferons in patients with seasonal allergic rhinitis. (a) The principal component analysis (PCA) plot gives an overview of the differentially expressed genes in patients before and one year after treatment as well as healthy controls. (b) The workflow of the selection of putative genes regulated by SLIT. (c) GO term enrichment of genes reacting to SLIT treatment using AMIGO. The numbers in the bars represent numbers of genes identified for each term. The *p* value comes from the comparison of patients before SLIT and after one year of SLIT in step 3 in (b). Red line is *p* value 0.05 (−log_10_ = 1.3).

Clinically, all of the patients in our study experienced improvement of their symptoms after the first and second years of treatment, based on evaluation by the same physician, which agrees with previous studies [13, 14]. In order to more comprehensively examine gene expression changes, we performed expression profiling studies of four healthy control and four paired patient samples, taken before and after one year of SLIT. Principal component analysis (PCA) based on the gene expression microarray data showed that the samples taken before treatment were grouped and clearly distinguished from the healthy controls, whereas the samples taken one year after the initiation of SLIT resembled the healthy controls more than the patients' own samples before treatment (Figure 1(a), ArrayExpress: A-MEXP-2320). The PCA result showed a clear difference between the patient samples before and the patient samples after treatment. It is possible that variations in age and gender could confound the result. However, inclusion of these two factors in the PCA plot did not support this possibility (supplementary Figure  1 and Table  1 in Supplementary Material available online at http://dx.doi.org/10.1155/2016/5153184).

To be able to clearly see the potential SAR-associated genes affected by SLIT a novel strategy to select genes from the gene expression microarray material for pathway analysis was designed (Figure 1(b)). The expression of genes reacting when challenged with allergen differed greatly, but not completely, between patients before treatment and healthy controls. This is in agreement with previous studies by us and others showing that CD4^+^ T cells from healthy controls also respond to allergen challenge [15, 16].

As outlined in Figure 1(b), our strategy is based on a series of steps to select putative genes regulated by SLIT. In step  1, we excluded genes that showed similar expression changes in response to allergen challenge in patients and controls.

In step  2, the genes whose expressions were significantly different in patients compared to healthy controls were selected to distinguish the genes which are putative SAR-associated genes. These genes contained three different categories: genes only responding to allergen in patients, genes only responding to allergen in controls, and genes responding to allergen in both patients and controls (but the response is significantly different) and all three are equally important. The genes which are silenced in patients but present in healthy controls are the genes that should be reacting in patients but for some reason do not.

In step 3, we eliminated the genes whose expression was not altered by SLIT, since we were interested in the treatments effect on the remaining genes. The final list consisting of putative genes regulated by SLIT underwent a GO biological processes enrichment analysis with AMIGO and was found to be enriched for several pathways connected to type I interferon signaling, namely, type I interferon signaling pathway (*p* = 2.05 × 10^−4^), cellular response to type I interferon (*p* = 2.05 × 10^−4^), and response to type I interferon (*p* = 2.29 × 10^−4^) (Figure 1(c) and supplementary Figures  2 and 3).

**Figure 2:**
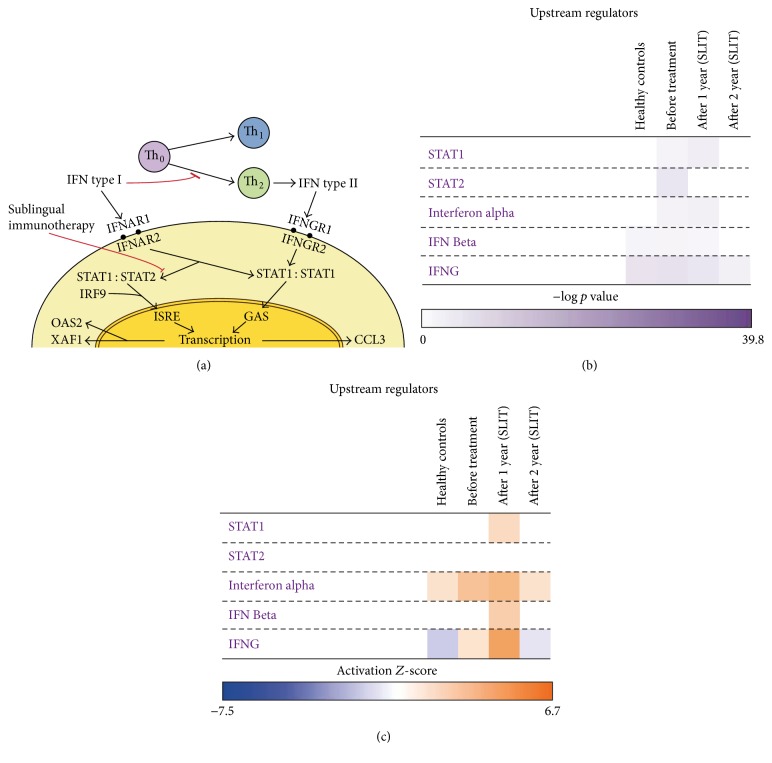
Sublingual immunotherapy causes complex changes of the key genes of the interferon pathway. (a) The diagrammatic sketch of potential involvement of type I interferons in T cell differentiation and sublingual immunotherapy. (b) The comparison of expression of upstream regulators,* STAT1, STAT2*,* IFNα*,* IFNβ,* and* IFNG,* in the diluent- and allergen-challenged cells of healthy controls and patients before treatment, after one year of SLIT, and after two years SLIT. Results are presented as –log (*p* value). The *p* value comes from the Fisher exact test done between the different comparisons for that particular gene significance. (c) The activation* Z*-score of expression of upstream regulators,* STAT1, STAT2*,* IFNα*,* IFNβ,* and* IFNG,* in the diluent- and allergen-challenged cells of healthy controls and patients before treatment, after one year of SLIT, and after two years of SLIT.

Type I interferons induce intracellular signaling via both the STAT1 : STAT2 heterodimer and the STAT1 : STAT1 homodimer [5], in addition to their ability to inhibit the differentiation of naive T cells to Th1 [5]. Signaling via the STAT1 : STAT1 homodimer can also be induced by type II interferons, especially IFN*γ*, which is commonly associated with Th1 cells [5] (Figure 2(a)). There are many type I IFNs, including IFN*α* and IFN*β*, and all type I IFNs bind a common cell-surface receptor, which is known as the type I IFN receptor [17–19]. IFN*γ* binds a different cell-surface receptor, which is known as the type II IFN receptor [20, 21].

For the further validation, we continued recruiting the same patient after the second year's therapy and did the same challenge and gene expression profiling but analyzed the high-throughput data using Ingenuity Pathways Analysis (IPA). Focusing on type I interferon pathway, the –log *p* value of the upstream regulators* STAT2* was differentially expressed in patients before treatment compared with healthy controls but showed no difference either one year or two years after the SLIT (Figure 2(b)). However,* STAT1* differed in expression in patients both before and after one year SLIT compared with controls, but not after two years (Figure 2(b)). A possible explanation is that* STAT2* is a part only of type I interferon pathway, while* STAT1* is a part in both type I and type II interferon pathways. The* IFNα* showed the same change as* STAT1 *(Figure 2(b)), which also supports that type I interferon pathway changed after SLIT, which may play an important role in the tolerance. However, the expression change of* IFNβ* and* IFNγ* was complex. Meanwhile in activation* Z*-score analysis, STAT2 had no significant change in all time points; STAT1 and* IFNβ* only changed significantly after one year of SLIT compared with the other time points;* IFNα* and* IFNγ* kept the same trend showed before and after one-year treatment were more different than after two-year treatment and controls (Figure 2(c)). Limited material size and variability in response to treatment are both possible explanations. Another possible explanation could be that the differentially expressed genes were identified based on our previously described criteria (a 1.5-fold change and *p* < 0.05). These criteria have been and are also used by many others but do not involve correction for multiple inference. On the other hand, pathway analysis showed statistically significant enrichment of the genes that were identified by those criteria. Finally, different programs for pathway analyses could affect the results. However, comparisons between different programs have shown that they have similar performance [22].

## 4. Conclusions

In summary, a decrease of interferon type I-mediated signaling is a possible cause of the shift from Th2 to Th1 in response to allergen in patients suffering from seasonal allergic rhinitis treated with sublingual immunotherapy; however, this requires more extensive investigations in larger materials.

## Supplementary Material

The supplementary material offers a table about the age and gender of the patients and controls and three figures to show that 1) the variations in age and gender did not confound the PCA result; 2) differentially expressed genes in controls and patients and the seven genes in interferon pathway showed that type I interferon signaling found to be enriched after SLIT treatment.

## Abbreviations

IFN*γ*: Interferon gamma
PBMCs: Peripheral blood mononuclear cells
PCA: Principal component analysis
SAR: Seasonal allergic rhinitis
SIT: Specific immunotherapy
SLIT: Sublingual specific immunotherapy
Th1: Type 1 T helper cell
Th2: Type 2 T helper cell
Tregs: T regular cells
IPA: Ingenuity Pathways Analysis.
