# Supplementary material for: Potential Involvement of Type I Interferon Signaling in Immunotherapy in Seasonal Allergic Rhinitis
Source: J Immunol Res. 2016 Dec 19;2016:5153184. doi: 10.1155/2016/5153184 (PMC5209614; doi:10.1155/2016/5153184)
Supplement: Supplementary file 1 — The supplementary material offers a table about the age and gender of the patients and controls and three figures to show that 1) the variations in age and gender did not confound the PCA result; 2) differentially expressed genes in controls and patients and the seven genes in interferon pathway showed that type I interferon signaling found to be enriched after SLIT treatment. [file 5153184.f1.docx]

**Supplementary files**

| Table 1. The age and gender of patients and controls | | |
| --- | --- | --- |
| ID | Gender | Age (years) |
| P1 | female | 50 |
| P2 | female | 44 |
| P3 | female | 36 |
| P4 | female | 65 |
| HC1 | female | 33 |
| HC2 | male | 33 |
| HC3 | male | 37 |
| HC4 | female | 33 |

**Supplementary figures**

**
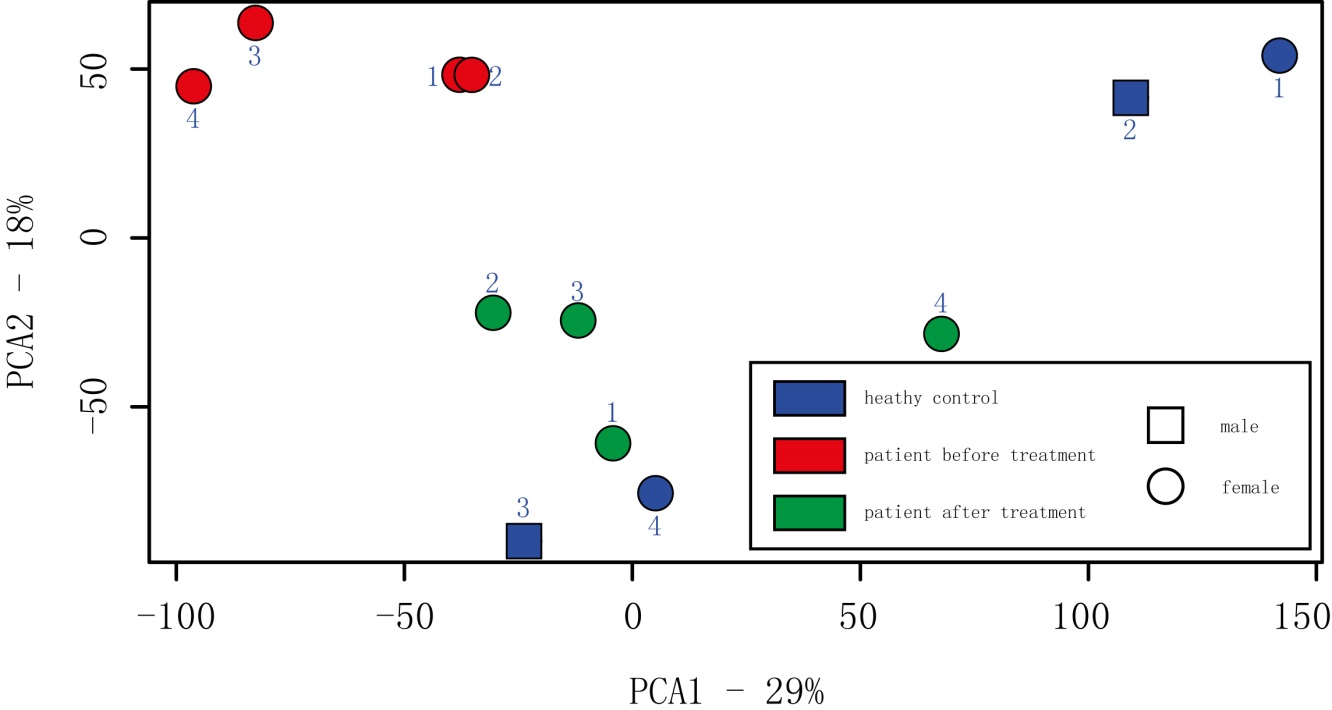
**

**Supplementary Figure 1: The Principal Component Analysis (PCA) plot of the differentially expressed genes in patients before and one year after treatment as well as healthy controls.** All male subjects were shown as square and females were round shape. Healthy controls were colored with blue, patients before treatment were colored with red and patients after one year SLIT were colored with green.

**
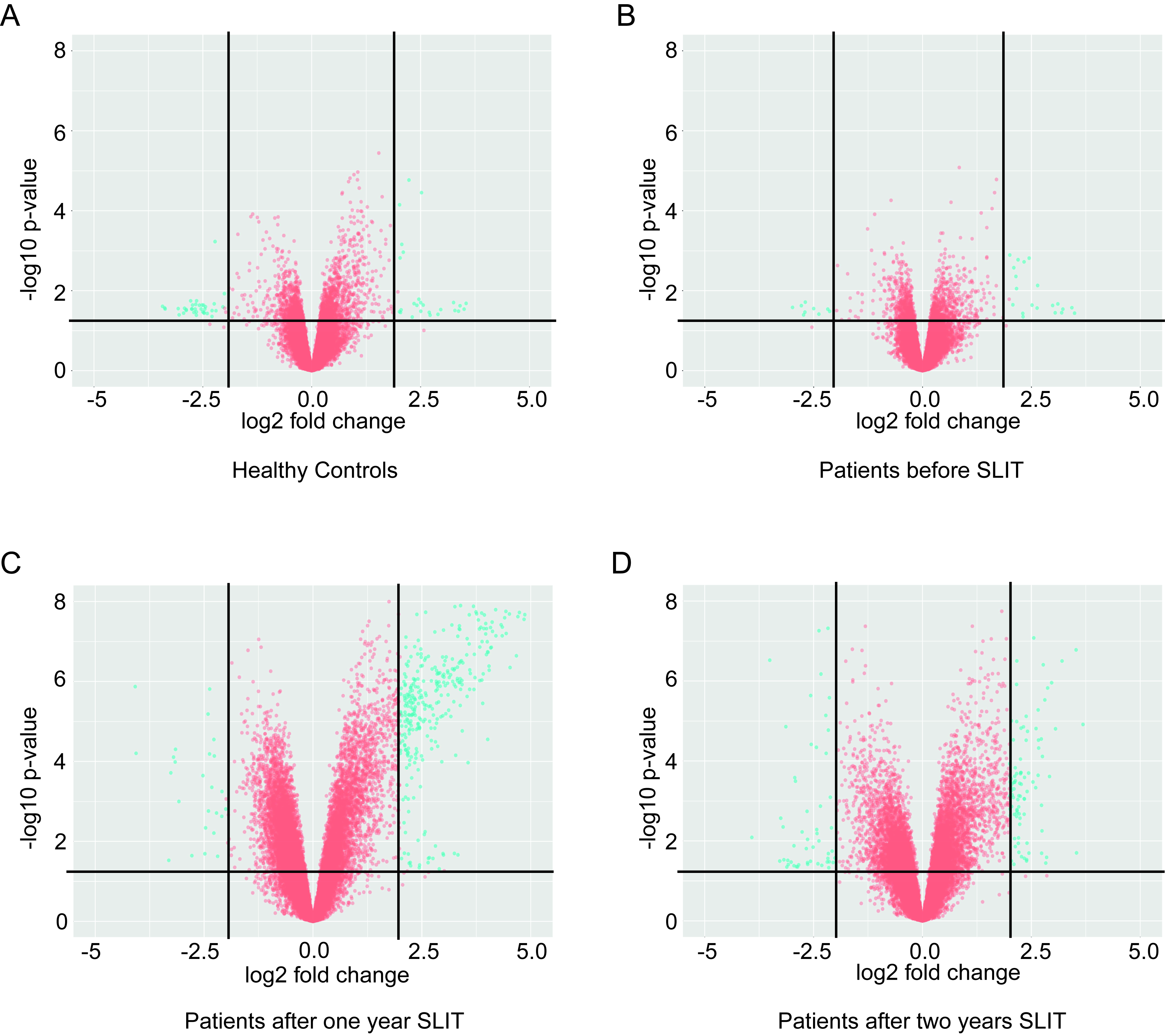
**

**Supplementary figure 2: The Volcano plot of differentially expressed genes in patients before, after one year SLIT and after two years SLIT as well as healthy controls. A.** healthy controls; **B.** patients before SLIT; **C.** patients after one year SLIT; **D.** patients after two years SLIT.

**
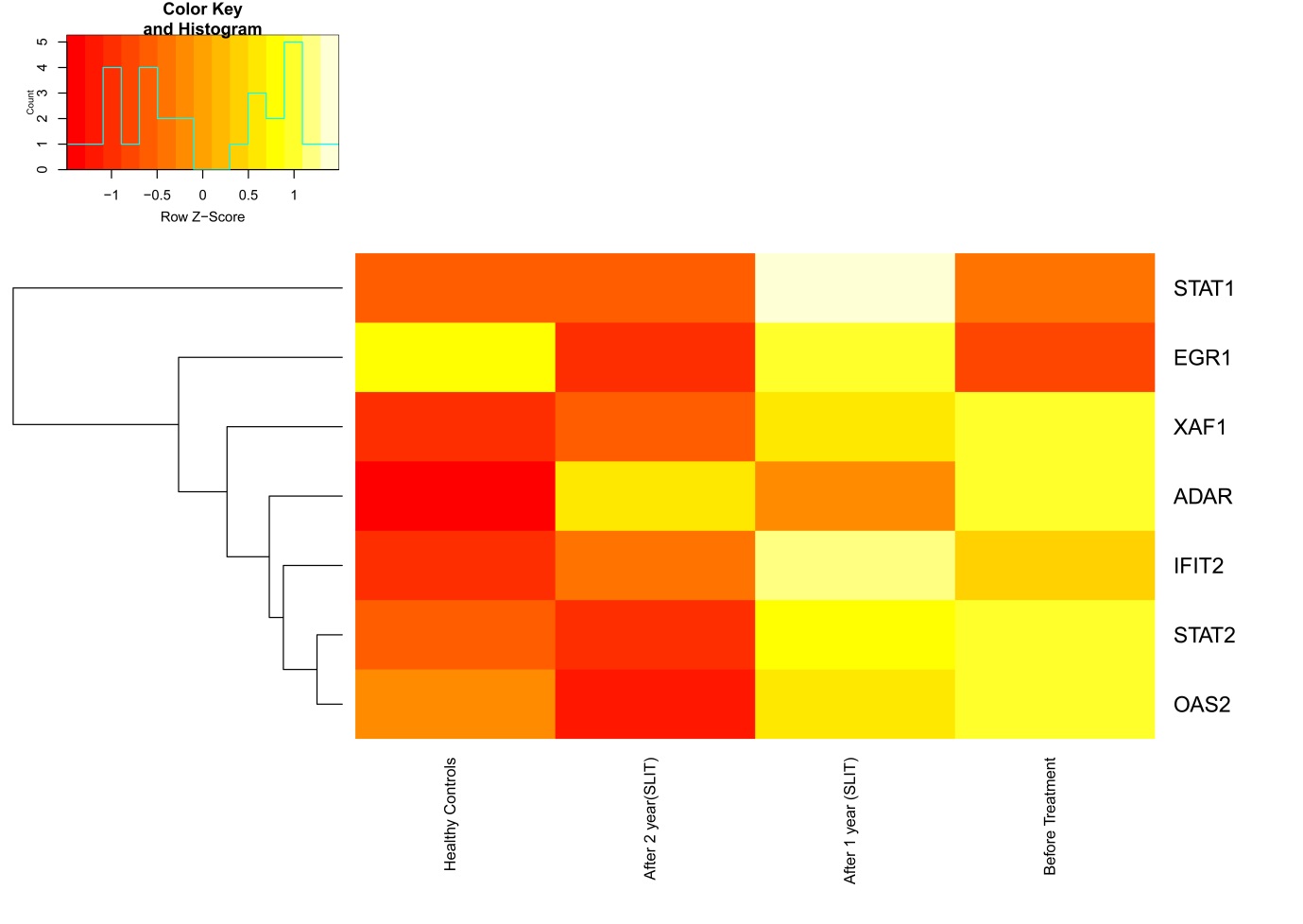
**

**Supplementary figure 3: Heat map of the expression of the seven genes in the interferon pathway.** The expression of *STAT1, EGR1, XAF1, ADAR, IFIT2, STAT2* and *OAS2* in healthy controls, patients before, after one year and after two years SLIT.
